# Intracorporeal Versus Extracorporeal Urinary Diversion Following Robotic-Assisted Radical Cystectomy for Bladder Cancer in Patients ≥ 65 Years of Age: A Systematic Review and Meta-Analysis

**DOI:** 10.7759/cureus.78406

**Published:** 2025-02-03

**Authors:** Konstantinos Kossenas, Riad A Kouzeiha, Olga Moutzouri, Filippos Georgopoulos

**Affiliations:** 1 Surgery, Nicosia Medical School, Nicosia, CYP; 2 Medical School, Lebanese University, Hadath Campus, Beirut, LBN; 3 Medical School, University of Nicosia, Nicosia, CYP; 4 Gastroenterology and Hepatology, Al Zahra Hospital, Dubai, ARE

**Keywords:** bladder tumour, minimally invasive laparoscopy, radical cystectomy, robot, urinary diversion

## Abstract

There is scarce information regarding intracorporeal (ICUD) and extracorporeal urinary diversion (ECUD) for the treatment of bladder cancer in patients aged 65 and older. This review aims to investigate this literature gap.

Following Preferred Reporting Items for Systematic Reviews and Meta-Analyses (PRISMA) criteria, this systematic review and meta-analysis was prospectively registered with PROSPERO (registration number CRD42024620211).

We searched PubMed, Scopus, and the Cochrane Library up to April 28, 2024, for any relevant literature comparing ICUD to ECUD in patients aged ≥65 years. We pooled the data using either weighted mean differences or odds ratios with random effects models. Heterogeneity was assessed with the Higgins I^2^ statistic, and the study quality was examined with the Newcastle-Ottawa scale. For results with moderate to high heterogeneity, we conducted a sensitivity analysis by excluding one study at a time. Nine studies with 4,340 patients in total, 1,967 in ICUD and 2,373 in ECUD, were included in the meta-analysis. The results showed that the ICUD significantly reduced the estimated blood loss (weighted mean differences (WMD): -64.34 mL, 95% CI: -113.26, -15.42; I² = 92%, p-heterogeneity < 0.00001, p-overall = 0.010), blood transfusion rates (OR: 0.29, 95% CI: 0.11, 0.76; I² = 86%, p-heterogeneity < 0.0001, p-overall = 0.01), and overall gastrointestinal complications (OR: 0.65, 95% CI: 0.46, 0.92; I² = 0%, p-heterogeneity = 0.70, p-overall = 0.02), when compared to ECUD, in patients 65 and over. However, we observed no significant differences, with regards to the operative duration (WMD: 3.48, 95% CI: -28.42, 35.38; I² = 98%, p-heterogeneity < 0.00001, p-overall = 0.83), length of hospitalization (WMD: 0.53, 95% CI: -0.15, 1.21; I² = 85%, p-heterogeneity < 0.00001, p-overall = 0.13), 30-day complication rates (OR: 1.22, 95% CI: 0.83, 1.78; I² = 77%, p-heterogeneity = 0.0005, p-overall = 0.30), 90-day complication rates (OR: 0.92, 95% CI: 0.61, 1.39; I² = 61%, p-heterogeneity = 0.04, p-overall = 0.68), or 30-day readmission rates (OR: 0.86, 95% CI: 0.62, 1.20; I² = 35%, p-heterogeneity = 0.20, p-overall = 0.38). The sensitivity analysis showed that several studies increased the heterogeneity, especially for results like the expected volume of blood loss and the transfusion rate. Overall, elderly patients undergoing robot-assisted radical cystectomy (RARC) may benefit from ICUD with regard to reduced blood loss, lower rates of blood transfusion, and decreased rate of gastrointestinal complications. However, more robust studies are required in order to reach firm findings.

## Introduction and background

Bladder cancer is a prevalent urinary system malignancy characterized by high rates of incidence and recurrence. Over the past few decades, its incidence has been on the rise [1] and the elderly population seems to be greatly affected [2,3]. Radical cystectomy is the main treatment option for patients with muscle-invasive bladder cancer [4]. Initially, open resections were the standard of surgical care; however, as the minimally invasive approaches and techniques progress in all fields of surgery [5-7], more patients undergo minimally invasive cystectomies. One such method is robot-assisted radical cystectomy (RARC), which may decrease overall morbidity and perioperative outcomes [8], particularly in older patients [9]. Moreover, cystectomies are inherently complicated as a procedure, with a crucial step for a successful cystectomy being the urinary diversion [10]. The standard approach has been the extracorporeal urinary diversion, in which surgeons bring the ureters and a section of the bowel outside the abdominal cavity, create the diversion, and then return the segment to the abdominal cavity. With the rise of RARC, an intracorporeal approach, namely intracorporeal urinary diversion (ICUD), has been proposed [11]. In this technique, surgeons perform the entire urinary diversion within the abdominal cavity, without exteriorizing any bowel segment. Unlike extracorporeal urinary diversion (ECUD), where the bowel is temporarily brought outside the abdomen for reconstruction, ICUD is performed robotically with minimal disruption to the bowel’s vascular supply and without additional incisions.

Given the potential evidence that RARC may offer better outcomes to elderly patients [12] along with the lack of research on RARC with ICUD in this age group, we believe that it is imperative to investigate whether RARC along with ICUD may offer greater benefits to patients 65 years of age or older as compared to ECUD. We thus aim to compare RARC ICUD to standard ECUD in this population, in terms of operative duration, intraoperative blood loss, blood transfusion rate, length of hospitalization, 30-day and 90-day complications, gastrointestinal complications, and readmission rates. With this meta-analysis, we aim to establish a comprehensive and solid understanding of the most safe, efficient, and effective type of urinary diversion in this age group. The results may assist surgeons in selecting the most appropriate approach for their patients and, by extension, improve surgical outcomes while enhancing their patients' recovery. Lastly, this meta-analysis may offer assistance in resource optimization and healthcare planning.

## Review

Materials and methods

We adhered to the Cochrane Collaboration’s standards for this systematic review and meta-analysis and complied with Preferred Reporting Items for Systematic Reviews and Meta-Analyses (PRISMA) to report our findings [13,14]. We prospectively registered our methodology for this review with PROSPERO (registration number: CRD42024620211).

Eligibility Criteria, Exclusion Criteria, and PICOTT

To determine which studies were eligible for inclusion, we followed the PICOTT (population, intervention, comparator, outcomes, types of study, time of follow-up) format. The population studied in this review (P) were patients 65 years of age or older who were diagnosed with muscle-invasive bladder cancer or high-risk non-muscle-invasive bladder cancer and had undergone robotic radical cystectomy as a treatment option. The intervention (I) was patients who had undergone ICUD, and the comparator (C) was patients who underwent ECUD. Interest-related outcomes included duration of surgery, length of hospital stay, predicted bleeding rates, estimated amount of blood transfused, gastrointestinal issues, total complications at 30 and 90 days, and 30-day readmission rates. Due to the inherent nature of the meta-analysis, we only considered primary studies, i.e. observation or randomized controlled trials (T). We excluded studies that did not align with our predefined PICOTT criteria, including those that deviated from the specified population (age < 65), intervention (single-arm/hybrid studies), comparison (other types of surgery), outcome, timing, and type of study (systematic reviews). Additionally, studies conducted on animal models, which inherently lack direct applicability to human clinical settings, were excluded. We allowed the follow-up time to be unconstrained since we primarily focused on perioperative outcomes. The last literature search was performed on April 28th, 2024.

Database Search

We searched the following three databases for relevant literature: PubMed, Scopus, and the Cochrane Library. Throughout the databases, the following search strategy was used: ("robot-assisted" OR robot OR robotic) AND ("bladder cancer" OR cystectomy) AND (intracorporeal OR extracorporeal OR "urinary diversion"). At this stage, no restrictions, such as age, were used. Once the articles were retrieved, two reviewers (KK, OM) independently, screened their tile and abstracts. Articles that met the inclusion criteria underwent full-text screening independently by the same reviewers. Once the articles to be included in this review were identified, we searched their reference lists for any potential articles that was missed during the preliminary search on the databases. During this process, conflicts or discrepancies were settled by agreement with the supervisor (FG).

Data Extraction

Microsoft Excel (Microsoft Corporation, Redmond, WA, US) was used to extract data. Two tables were created, one related to the study and patients’ characteristics, i.e. study author, design, country, number of patients in each group, total sample size, mean age and BMI, type of urinary diversion, and study period and the second that focused on the outcomes of interest such as operative duration, estimated blood loss, blood transfusion rate, length of hospital stay, overall complications at 30 and 90 days, gastrointestinal complications, and 30-day readmission rates. Since less than 10 studies were retrieved, each reviewer (KK, OM) performed data extraction separately and then the results were cross-checked to ensure that no mistakes took place. It was not necessary to make any assumptions or simplifications regarding data when performing data extraction. After retrieving all relevant information from the articles, it was not required to contact any of the authors for clarifications or additional data.

Assessment of Study Quality and Statistical Analysis

We employed the Newcastle-Ottawa scale (NOS) to evaluate the caliber of the included studies at the study level. Two reviewers (KK, RK) performed an assessment of study quality, independently. To analyze continuous outcomes, the inverse variance and weighted mean differences (WMD) with random effects models were utilized, and for dichotomous outcomes, the odds ratio (OR) with random effects models, using Mantel-Haenszel's method, was used. The random effects model over the fixed effects was used because the random effects model accounts for variability between studies and assumes different true effects sizes across them. If studies reported their outcomes with either medians with ranges or interquartile ranges (IQRs), they were converted to means and standard deviations (SD) using the methods described by Wan et al. [15]. The Higgins' I² statistic was used to determine the level of heterogeneity. We considered 0-40% as possibly not important, 30-60% as a moderate level of heterogeneity, 50-90% as substantial, and 75-100% as a considerable degree of heterogeneity, as described by the Cochrane Handbook [16]. A value of p<0.05 was considered statistically significant. Moreover, the chi² statistic was also calculated, with p<0.10 signifying significant heterogeneity. Tau² values were used to assess variance. For results with moderate to high heterogeneity, a sensitivity analysis was conducted by “leaving one out” or removing one study at a time, to determine studies that added disproportionately to the observed variability. Since we retrieved less than 10 studies from the literature search, we did not assess publication bias via funnel plots. Lastly, we used Cochrane Review Manager (RevMan) software [17] to perform the statistical analysis and to visualize the pooled effects. All data were retrieved from the papers and we did not have to contact the authors.

Results

Screening Results

**Figure 1:**
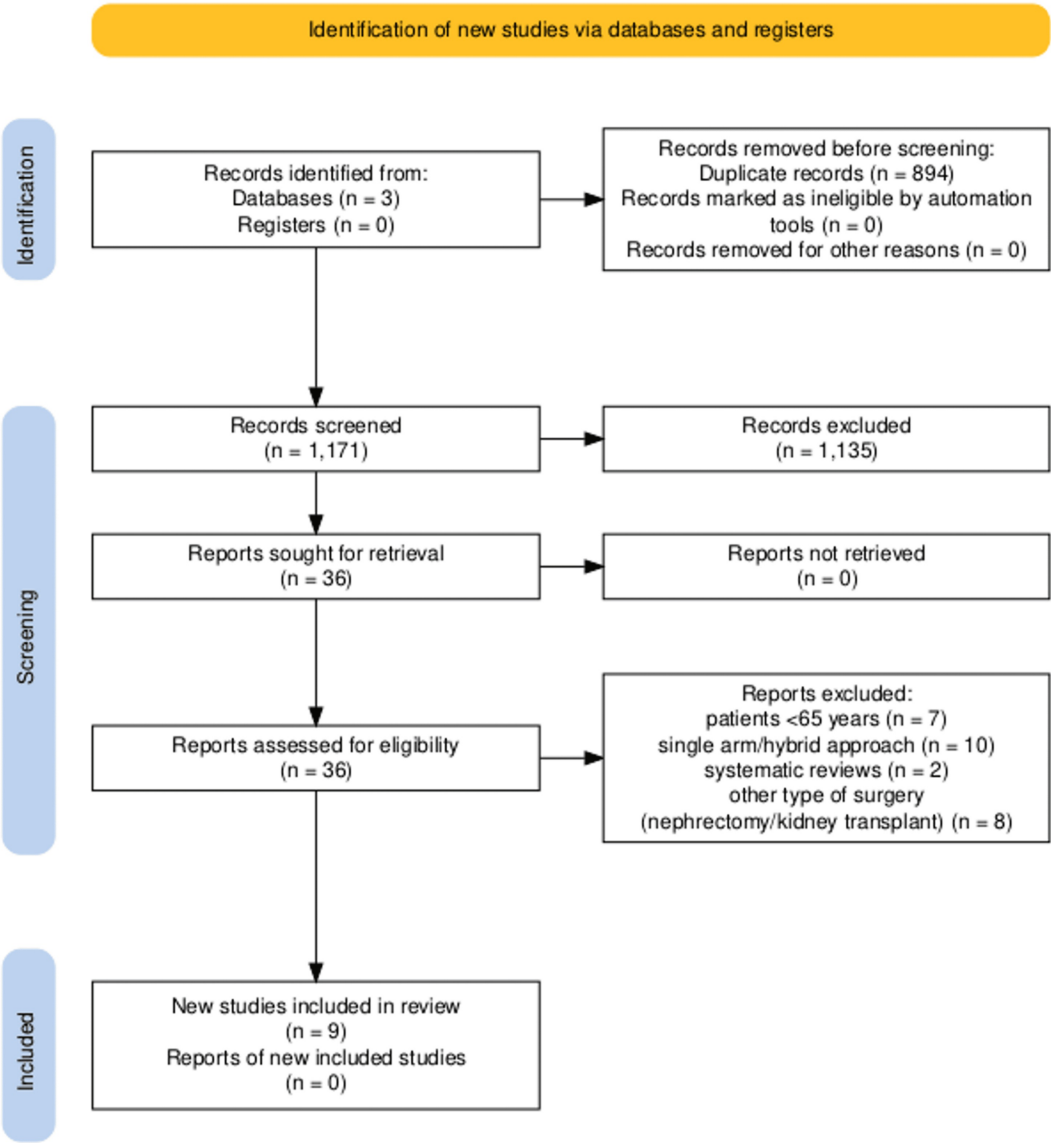
PRISMA 2020 flowchart PRISMA: Preferred Reporting Items for Systematic Reviews and Meta-Analyses

From the database search, a total of 2,065 papers were retrieved; 809 from PubMed, 1,101 from Scopus, and 155 from the Cochrane Library. We imported the papers into Rayyan software [18] and identified 894 papers as duplicates. Two reviewers (KK, OM) independently screened the duplicates and removed them. For the remaining 1,171 articles, two reviewers screened their titles and abstracts and determined that 36 were eligible for full-text review. From the 36 articles, a total of 9 studies were selected for data extraction. Figure 1 shows the Preferred Reporting Items for Systematic Reviews and Meta-Analyses (PRISMA) flowchart that details the screening procedure.

Study and Patient Demographics

Nine studies were used to retrieve data, with a total of 4340 patients: 1967 in ICUD and 2373 in ECUD. Two studies were conducted in the UK, three studies in the US, one in Denmark, one in France, and one in Japan [19-27]. All studies were retrospective in nature, with the exception of two studies that were prospective [20,23]. The sample size ranged from 13 to 1094 patients in the ICUD arm and from 13 to 1031 patients in the ECUD arm. All patients were above 65, with the highest age being 71 for ICUD and 73 for ECUD. A variety was also observed with regard to BMI, with many arms having overweight patients. The urinary diversion type was either ileal conduit or neo-bladder construction, with the ileal conduit being more frequently observed. Lastly, the study periods ranged from 2003 all the way to 2021 (Table 1).

**Table 1:** Baseline characteristics of the included studies RCS: retrospective cohort study, PCS: prospective cohort study, ICUD: intracorporeal urinary diversion, ECUD, extracorporeal urinary diversion, UD: urinary diversion, IC: ileal conduit, NB: neobladder, n/a: not applicable, NOS: Newcastle-Ottawa scale

Study	Country	Design	Sample size (ICUD/ECUD)	Age (ICUD/ECUD)	BMI (ICUD/ECUD)	UD type (ICUD/ECUD)	Study period (ICUD/ECUD)	NOS
Ahmed et al. 2014 [19]	UK	RCS	167/768	66/68	28/27	IC/NB	2003-11	8
Bertolo et al.2019 [20]	US	PCS	60/66	69/73	n/a	IC	2014-17	8
Guru et al. 2010 [21]	US	RCS	13/13	71/66	n/a	IC	2005-9	8
Hussein et al. 2018 [22]	US	RCS	1094/1031	67/68	27.3/27.5	IC/NB	2005-16	8
Kingo et al. 2017 [23]	Denmark	PCS	38/12	68/68	27.3/24.3	IC	2012-15	7
Lenfant et al. 2018 [24]	France	RCS	74/34	65/68	25.9/26.2	IC/NB	2010-16	7
Morizane et al. 2024 [25]	Japan	RCS	155/150	71/71	22.6/22.8	NB	2018-21	8
Tan et al. 2019 [26]	UK	RCS	59/50	71/69	26.5/27	IC	2015-2017	8
Teoh et al. 2021 [27]	China	RCS	307/249	66/68	24.47/24.32	IC/NB	2007-2020	7

Operative Outcomes

Seven studies examined the operative duration, with inconclusive evidence on either approach’s efficiency [19,20,22-24,26,27]. Eight studies [20-27] examined the intra-operative estimated blood loss and all apart from Bertolo et al. [20] favor ICUD over ECUD. Four studies examined the blood transfusion rate and the majority showed a decreased rate in patients receiving ICUD [20,22,24,25]. Eight studies examined the length of hospitalization with inconclusive evidence [19,20,22-27]. Mixed results were also observed for both approaches with regard to 90- and 30-day complication rates and 30-day readmission rates. For ICUD, a decreased incidence of gastrointestinal issues was noted (Table 2).

**Table 2:** Operative outcomes of ICUD vs ECUD OD: operative duration, EBL: estimated blood loss, BTR: blood transfusion rate, LOH: length of hospitalization, 30Dc: 30 days complications rate, 90Dc: 90 days complications rate, GIc: gastrointestinal complications, 30Dr: 30 days readmission rate, ICUD: intracorporeal urinary diversion, ECUD: extracorporeal urinary diversion, n/a: not available

Study	OD (±SD)	EBL	BTR	LOH	30Dc	90Dc	GIc	30Dr
	ICUD/ECUD	ICUD/ECUD	ICUD/ECUD	ICUD/ECUD	ICUD/ECUD	ICUD/ECUD	ICUD/ECUD	ICUD/ECUD
Ahmed et al. 2014 [19]	420±60/ 360±60	n/a	n/a	9.7±4.45/9.05±5.19	67/269	78/309	19/142	10/95
Bertolo et al.2019 [20]	390.85± 84.75/ 386.54±63.78	380±380/ 350±300	8/6	7±5/8±4	13/9	2/4	5/10	7/7
Guru et al. 2010 [21]	n/a	314.58±258.29/453.85± 333.09	n/a	n/a	n/a	n/a	n/a	n/a
Hussein et al. 2018 [22]	357±30.75/ 400±35.5	300±98.75/350±87.5­­­­­	50/135	9±1.5/8±1.75	335/195	50/40	n/a	57/56
Kingo et al. 2017 [23]	311.05±71.05/ 332.67±79.14	185.42±213.91/524.18±519	n/a	10.89±9.23/8.17±1.47	n/a	n/a	5/1	n/a
Lenfant et al. 2018 [24]	320±30/ 285±20	400±87.5/500±112.5	4/7	14±2/12±2	37/13	n/a	n/a	n/a
Morizane et al. 2024 [25]	n/a	370.9±472.5/222.8± 401.25	15/80	22.6±37.75/21.9±19	n/a	61/81	n/a	n/a
Tan et al. 2019 [26]	330±17/ 375±26.75	300±50/425±50	n/a	8±1.25/8±0.75	30/50	7/10	n/a	n/a
Teoh et al. 2021 [27]	362.8±94.9/ 329.38±147.81	423.08± 361.12/541.3±474.32	n/a	15.7±12.25/17.81±11.61	157/118	n/a	27/28	72/58

Risk of Bias

We used the NOS to evaluate the included studies across three main domains: selection, comparability, and outcome/exposure. The selection domain examines the representativeness of the population studied and its exposure and baseline characteristics (4 points). The comparability domain examines how well its study accounts for its confounders (2 points). The outcome domain examined the adequacy of the follow-up duration, the methods used to assess the outcomes, and the reliability of the response (3 points). In total, nine points can be awarded, with the higher score reflecting a higher study quality. In this systematic review, three studies scored 7 points each with the remaining scoring 8 [23,24,27]. Overall, the studies showed high methodological quality (Appendices)

Meta-analysis

The results of the operative duration, intra-operative estimated blood loss, blood transfusion rate, length of hospitalization, overall complications at 30 days, overall complications at 90 days, overall gastrointestinal complications, and readmission within 30 days were analyzed.

Operative Duration

**Figure 2:**
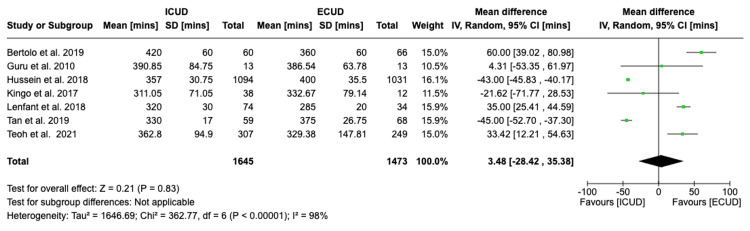
Forest plot of operative duration Source: Bertolo et al. [20], Guru et al. [21], Hussein et al. [22], Kingo et al. [23], Lenfant et al. [24], Morizane et al. [25], Tan et al. [26], Teoh et al. [27]

Seven studies with a total of 3,118 patients (1,645 in ICUD and 1,473 in ECUD) examined the operative duration and concluded a non-significant association between the two approaches [20-24,26,27]. The details are: WMD: 3.48 (95%CI -28.42, 35.38), I² = 98%, P-heterogeneity < 0.00001, P-overall = 0.83 (Figure 2).

Estimated Intraoperative Blood Loss

**Figure 3:**
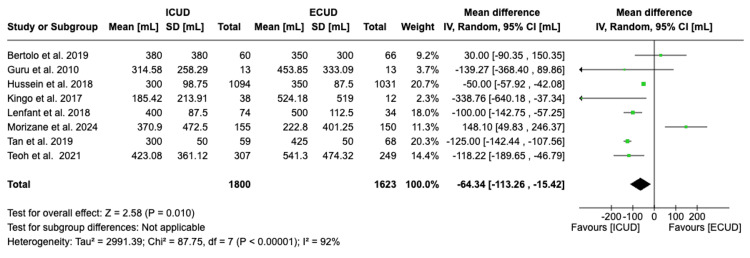
Forest plot of estimated blood loss Source: Bertolo et al. [20], Guru et al. [21], Hussein et al. [22], Kingo et al. [23], Lenfant et al. [24], Morizane et al. [25], Tan et al. [26], Teoh et al. [27]

Eight studies examined the estimated intraoperative blood loss in a total of 3,423 patients (1,800 in ICUD and 1,623 in ECUD) [20-27]. A significant decrease in blood loss of 64.34 mL was observed in favor of ICUD. The details are: WMD: -64.34 (95%CI -113.26, -15.42), I² = 92%, P-heterogeneity < 0.00001, P-overall = 0.010 (Figure 3).

Blood Transfusion Rate

**Figure 4:**
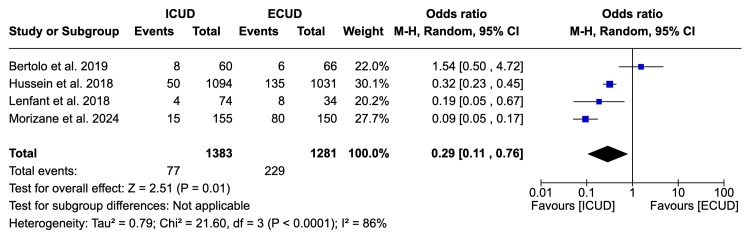
Forest plot of blood transfusion rate Source: Bertolo et al. [20], Hussein et al. [22], Lenfant et al. [24], Morizane et al. [25]

Four studies examined the blood transfusion rate in a total of 2,664 patients (1,383 in ICUD and 1,281 in ECUD) [20,22,24,25]. A significant associated decrease of approximately 70% was observed in favor of ICUD. The details are: OR: 0.29 (95%CI 0.11, 0.76), I² = 86%, P-heterogeneity < 0.0001, P-overall = 0.01 (Figure 4).

Length of Hospitalization

**Figure 5:**
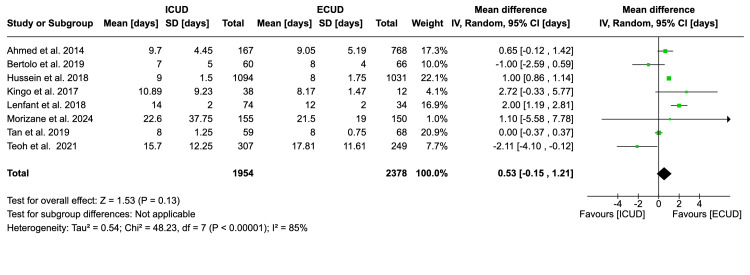
Forest plot of the length of hospitalization Source: Ahmed et al. [19], Bertolo et al. [20], Guru et al. [21], Hussein et al. [22], Kingo et al. [23], Lenfant et al. [24], Morizane et al. [25], Tan et al. [26], Teoh et al. [27]

Eight studies examined the length of hospitalization in a total of 4,332 patients (1,954 in ICUD and 2,378 in ECUD) [19,20,22-27]. A non-significant association was observed between the two approaches. The details are: WMD: 0.53 (95%CI -0.15, 1.21), I² = 85%, P-heterogeneity < 0.00001, P-overall = 0.13 (Figure 5).

Thirty-Day Complication Rate

**Figure 6:**
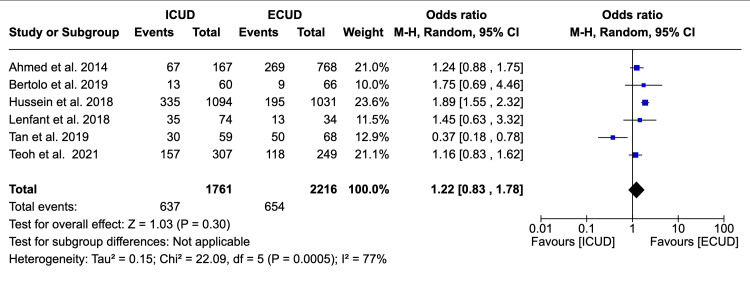
Forest plot of 30-day complication rates Source: Ahmed et al. [19], Bertolo et al. [20], Hussein et al. [22], Lenfant et al. [24], Tan et al. [26], Teoh et al. [27]

Six studies examined the 30-day complication rate in a total of 3,977 patients (1,761 in ICUD and 2,216 in ECUD) [19,20,22,24,26,27]. A non-significant association was observed between the two approaches. The details are: OR: 1.22 (95%CI 0.83, 1.78), I² = 77%, P-heterogeneity = 0.0005, P-overall = 0.30 (Figure 6).

Ninety-Day Complication Rate

**Figure 7:**
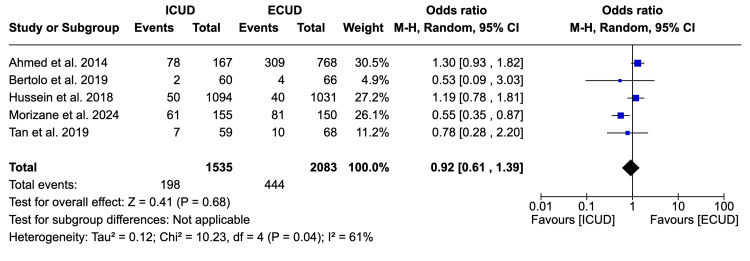
Forest plot of 90-day complication rates Source: Ahmed et al. [19], Bertolo et al. [20], Hussein et al. [22], Morizane et al. [25], Tan et al. [26]

Five studies [19,20,22,26] examined the 90-day complication rate in a total of 3,618 patients (1,535 in ICUD and 2,083 in ECUD). A non-significant association was observed between the two approaches. The details are: OR: 0.92 (95%CI 0.61, 1.39), I² = 61%, P-heterogeneity = 0.04, P-overall = 0.68 (Figure 7).

Gastrointestinal Complication Rate

**Figure 8:**
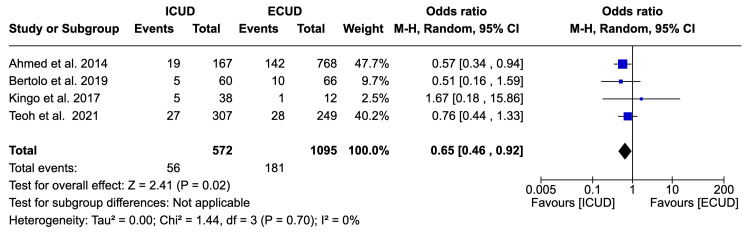
Forest plot of gastrointestinal complication rates Ahmed et al. [19], Bertolo et al. [20], Kingo et al. [23], Teoh et al. [27].

Four studies examined the gastrointestinal complication rate in a total of 1,667 patients (572 in ICUD and 1,095 in ECUD) [19,20,23,27]. A significant decrease of 35% was observed in favor of ICUD. The details are: OR: 0.65 (95%CI 0.46, 0.92), I² = 0%, P-heterogeneity = 0.70, P-overall = 0.02 (Figure 8).

Thirty-Day Readmission Rate

**Figure 9:**
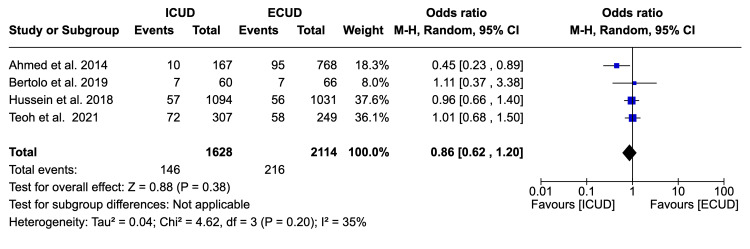
Forest plot of 30-day readmission rates Source: Ahmed et al. [19], Bertolo et al. [20], Hussein et al. [22], Teoh et al. [27]

Four studies examined the 30-day readmission rate in a total of 3,742 patients (1,628 in ICUD and 2,114 in ECUD) and observed a non-significant association between the two approaches [19,20,22,27]. The details are: OR: 0.86 (95%CI 0.62, 1.20), I² = 35%, P-heterogeneity = 0.20, P-overall = 0.38 (Figure 9).

Sensitivity Analysis With “Leave One Out”

We conducted a sensitivity analysis by eliminating one study at a time for results with moderate to high heterogeneity, to determine any study that adds substantially to variability observed. It revealed that most studies accounted for the variability observed in the outcomes. However, certain studies significantly influenced the results. For instance, excluding Hussein et al. [22], Lenfant et al. [24], and Tan et al. [26] rendered the estimated blood loss results non-significant while excluding three studies [22,24,25] had a similar effect on blood transfusion rates. Length of hospitalization results became significant in favor of ECUD when studies by Bertolo et al. [20] and Teoh et al. [27] were excluded. For the 30-day complication rate, excluding Tan et al. [26] increased the rate significantly in favor of ECUD. Additionally, heterogeneity in the 90-day complication rate dropped to 0% when the study by Morizane et al. [25] was excluded, highlighting its substantial contribution to variability. Similarly, heterogeneity for the 30-day readmission rate dropped to 0% upon excluding Ahmed et al. [19], indicating its significant impact. These findings underscore the importance of individual studies in shaping the pooled results and heterogeneity across analyses (Table 3).

**Table 3:** Sensitivity analysis with the “leave one out” approach WMD=weighted mean difference, OR=odds ratio, ICUD=intracorporeal urinary diversion, ECUD=extracorporeal urinary diversion

Study excluded	WMD/OR (95%CI)	I^2^%	P-heterogeneity value	P-overall value	Comments
Operative duration					
Bertolo et al.2019 [20]	-6.61 (-38.50, 25.28)	98	<0.00001	0.68	All studies accounted for the variability observed
Guru et al. 2010 [21]	3.38 (-30.45, 37.22)	99	0.00001	0.84	
Hussein et al. 2018 [22]	11.95 (-31.99, 55.89)	98	0.00001	0.59	
Kingo et al. 2017 [23]	6.76 (-27.38, 40.89)	99	0.00001	0.70	
Lenfant et al. 2018 [24]	-3.00 (-29.96, 23.96)	96	0.00001	0.83	
Tan et al. 2019 [26]	12.18 (-34.27, 58.64)	99	0.00001	0.61	
Teoh et al. 2021 [27]	-1.83 (-35.64, 31.99)	98	0.00001	0.92	
Estimated blood loss					
Bertolo et al.2019 [20]	-73.92 (-125.26, -22.57)	93	< 0.00001	0.005	When excluding Hussein et al. [22], Lenfant et al. [24], and Tan et al [26], the results became non-significant
Guru et al. 2010 [21]	-61.36 (-111.45, -11.27)	93	<0.00001	0.02	
Hussein et al. 2018 [22]	-66.08 (-132.64, 0.47)	84	0.00001	0.05	
Kingo et al. 2017 [23]	-57.99 (-107.01, -8.98)	93	0.00001	0.02	
Lenfant et al. 2018 [24]	-56.10 (-112.45, 0.25)	93	0.00001	0.05	
Morizane et al. 2024 [25]	-91.07 (-138.86, -43.27)	91	< 0.00001	0.0002	
Tan et al. 2019 [26]	-48.78 (-106.47, 8.92)	80	< 0.0001	0.10	
Teoh et al. 2021 [27]	-55.09 (-108.61, -1.56)	93	0.00001	0.04	
Blood transfusion rate					
Bertolo et al.2019 [20]	0.18 (0.07, 0.45)	83	0.003	0.0002	When excluding three studies [22,24,25], the results became non-significant.
Hussein et al. 2018 [22]	0.29 (0.05, 1.63)	89	0.0001	0.16	
Lenfant et al. 2018 [24]	0.33 (0.10, 1.07)	91	0.0001	0.06	
Morizane et al. 2024 [25]	0.44 (0.16, 1.24)	75	0.02	0.12	
Length of hospitalization					
Ahmed et al. 2014 [19]	0.49 (-0.31, 1.29)	87	0.00001	0.23	Results became significant in favor of ECUD when two studies [20,27] were excluded.
Bertolo et al.2019 [20]	0.70 (0.01, 1.40)	86	0.00001	0.05	
Hussein et al. 2018 [22]	0.36 (-0.62, 1.34)	81	0.0001	0.47	
Kingo et al. 2017 [23]	0.44 (-0.25, 1.13)	87	0.00001	0.22	
Lenfant et al. 2018 [24]	0.24 (-0.50, 0.97)	85	0.00001	0.53	
Morizane et al. 2024 [25]	0.52 (-0.17, 1.21)	88	0.00001	0.14	
Tan et al. 2019 [26]	0.66 (-0.13, 1.45)	74	0.0007	0.10	
Teoh et al. 2021 [27]	0.75 (0.09, 1.41)	85	0.00001	0.03	
30 days complication rate					
Ahmed et al. 2014 [19]	1.20 (0.73, 1.96)	81	0.0003	0.48	When excluding Tan et al. [26], the rate of complication in 30 days increased significantly in favor of ECUD.
Bertolo et al.2019 [20]	1.17 (0.77, 1.77)	82	0.0002	0.47	
Hussein et al. 2018 [22]	1.07 (0.72, 1.59)	60	0.04	0.73	
Lenfant et al. 2018 [24]	1.19 (0.78, 1.81)	82	0.0002	0.43	
Tan et al. 2019 [26]	1.46 (1.13, 1.90)	52	0.08	0.004	
Teoh et al. 2021 [27]	1.22 (0.75, 1.98)	80	0.0006	0.42	
90 days complication rate					
Ahmed et al. 2014 [19]	0.79 (0.48, 1.29)	50	0.11	0.34	When excluding Morizane et al. [25], the heterogeneity dropped to 0%, showing that this study contributed substantially to observed variability.
Bertolo et al.2019 [20]	0.94 (0.61, 1.46)	69	0.02	0.79	
Hussein et al. 2018 [22]	0.81 (0.45, 1.46)	68	0.02	0.69	
Tan et al. 2019 [26]	0.93 (0.58, 1.49)	70	0.02	0.76	
Morizane et al. 2024 [25]	1.20 (0.93, 1.54)	0	0.63	0.16	
30 days readmission rate					
Ahmed et al. 2014 [19]	0.99 (0.76, 1.29)	0	0.96	0.93	When excluding Ahmed et al. [19], the heterogeneity dropped to 0%, showing that the study contributed substantially to the observed heterogeneity.
Bertolo et al.2019 [20]	0.83 (0.55, 1.23)	55	0.11	0.35	
Hussein et al. 2018 [22]	0.79 (0.44, 1.40)	54	0.11	0.41	
Teoh et al. 2021 [27]	0.77 (0.45, 1.32)	50	0.14	0.34	

Discussion

Our goal in this meta-analysis was to compare ICUD to the conventional ECUD in elderly patients undergoing RARC, an underrepresented group in the literature. Our meta-analysis showed that ICUD offered reduced blood loss (WMD: -64.34 mL; 95% CI: -113.26, -15.42), reduced blood transfusion rates (OR: 0.29; 95% CI: 0.11, 0.76), and a significant 35% reduction in gastrointestinal complications (OR: 0.65; 95% CI: 0.46, 0.92) when compared to ECUD. However, no significant differences were observed between ICUD and ECUD regarding operative duration, length of hospitalization, 30-day, or 90-day complication rates and readmission rates. High heterogeneity was observed in multiple outcomes and our sensitivity analysis showed that some studies influence the observed heterogeneity in some outcomes. For instance, removing some studies rendered our results of blood loss [22,24,26] and transfusion rates [22,24,25] non-significant, whereas excluding some studies in the length of hospitalization [20,27] and 30-day complication rate [26] rendered our results significant in favor of ECUD. Additionally, removing certain studies, particularly in 90-day complications [25] and 30-day readmission [19], dropped heterogeneity to zero, indicating a substantial effect on the observed variability. Moreover, removing certain studies in the sensitivity analysis reduced heterogeneity significantly in 90-day complications [25] and 30-day readmissions [19], underscoring the impact of individual studies on overall findings.

Previous studies have aimed to compare ICUD to ECUD in patients undergoing RARC. However, the majority of them used a range of ages and did not focus primarily on the elderly, limiting their applicability to this age group. Pruthi et al. showed that ICUD could potentially lead to decreased blood loss and overall complication rates when compared to ECUD, partially aligning with our results of decreased blood loss and decreased gastrointestinal complications [28]. Kang et al. observed longer operative duration in ICUD compared to ECUD but no differences in other operative outcomes. However, our results contradict that of Kang et al. [29], showing no significant differences in the operative duration, further highlighting that ICUD may not offer the same advantages to the elderly. Our meta-analysis, however, agreed with the results of Pyun et al. [30], who concluded a significantly lower blood loss in the ICUD compared to ECUD. Shim et al. observed fewer complications and a shorter recovery period with ICUD, however, the younger patients included in their study may have influenced the recovery periods, compared to our elderly-focused study [31]. Mistretta et al. observed no major differences in both complication rates and functional outcomes, disagreeing with our study of reduced blood loss and gastrointestinal complications [32]. Lastly, Dalimov et al. found higher admission rates [33], contradicting our findings of no significant differences. The variations that were observed in the literature underscore the important role that age-specific analysis plays when comparing surgical approaches, particularly ICUD to ECUD for a complex surgery such as RARC.

Even though this systematic review and meta-analysis provides insights into a major surgery, it is still characterized by several limitations. First of all, most of the research that was incorporated into the synthesis was retrospective, which inherently is linked to a greater chance of bias than randomized clinical trials or prospective studies. This, to some extent, limits the ability to establish causal relationships between the ICUD and the outcomes. In addition, we observed significant heterogeneity across the included studies. This potentially reflects a number of factors that were not accounted for in the primary research, such as the surgeon’s expertise and experience level, different institutional protocols, patients’ selection criteria, and a non-standardized data collection approach, leading to variability in the documented outcomes among the studies. Also, the small sample sizes observed in some studies limit the statistical power to identify differences in certain outcomes with a lower number of included studies, i.e., readmission rates; in addition, we cannot examine publication bias. Further, different types of diversions, i.e., ileal conduit versus neobladder, may have affected several outcomes, including operative duration, blood loss, etc., and need to be standardized in future studies. Therefore, it is advised to evaluate the review’s findings cautiously, especially if they are to be applied in clinical practice.

Overall, the results of this meta-analysis demonstrate some substantial advantages of ICUD compared to ECUD in elderly patients undergoing RARC, particularly, reduced blood loss, blood transfusion rate, and overall gastrointestinal complications. These findings support the incorporation of ICUD into clinical practice when appropriate patients are identified. However, the surgeon’s expertise and patient-specific considerations need to be incorporated into a shared decision-making process. Policymakers should potentially focus on investing in robotic surgical training for surgeons interested in acquiring the necessary skills to perform the ICUD to increase patient accessibility to ICUD. Since robotic approaches are on the rise, and likely, the robotic system will become the standard of surgical care in many fields [34,35], future studies aiming to compare ICUD to ECUD in RARC must be prospective in nature, particularly randomized, to increase the generability of the findings as well as include a long follow up duration, to capture outcomes currently not available in the literature, such as five-year mortality of disease-free survival. Also, future studies might need to perform subgroup analysis to account for current gaps, i.e., neobladder versus ileal conduit to further refine the patient selection process. Lastly, evaluating the cost-effectiveness of ICUD compared to ECUD will assist policymakers, in optimizing healthcare resources and personnel allocation, to further improve outcomes for elderly patients.

## Conclusions

This systematic review and meta-analysis was the first one evaluating ICUD to ECUD in elderly patients undergoing RARC and concluded a significant decrease in blood loss, blood transfusion rate, and overall gastrointestinal complications in patients undergoing ICUD. There were no discernible variations between the two approaches with regard to operative duration, length of hospitalization, 30- and 90-day overall complications, and 30-day readmission rate. Limitations such as retrospective study designs and heterogeneity warrant caution when interpreting the results. Future research should focus on prospective studies and cost-effectiveness analyses to further validate these findings, optimize patient outcomes, identify patient subgroups, and further refine the patient selection process.

## Appendices

**Table 4:** Newcastle-Ottawa scale The Newcastle-Ottawa Scale (NOS) is a tool used to assess the quality of non-randomized studies, particularly cohort and case-control studies. It evaluates studies based on three main categories: Selection: How participants were selected (e.g., representativeness of the sample, adequacy of controls). Comparability: How comparable the groups are (e.g., control for confounding factors). Outcome: The assessment of outcomes (e.g., how outcomes are measured and the length of follow-up).

Study	Selection	Comparability	Outcomes	Overall
Ahmed et al. 2014 [19]	★★★★	★★	★★	8
Bertolo et al.2019 [20]	★★★★	★★	★★	8
Guru et al. 2010 [21]	★★★★	★★	★★	8
Hussein et al. 2018 [22]	★★★★	★★	★★	8
Kingo et al. 2017 [23]	★★★★	★★	★	7
Lenfant et al. 2018 [24]	★★★★	★★	★	7
Morizane et al. 2024 [25]	★★★★	★★	★★	8
Tan et al. 2019 [26]	★★★★	★★	★★	8
Teoh et al. 2021 [27]	★★★★	★★	★	7
